# Multiple Sclerosis, Melatonin, and Neurobehavioral Diseases

**DOI:** 10.3389/fendo.2017.00280

**Published:** 2017-10-23

**Authors:** Richard Wurtman

**Affiliations:** ^1^Massachusetts Institute of Technology, Cambridge, MA, United States

**Keywords:** melatonin, multiples sclerosis, light, darkness, seasonal affective disorder syndrome, neurodegeneration

## Introduction

Numerous neurobehavioral diseases typically exhibit annual rhythms in the frequency with which they cause flare-ups. A prime example is the seasonal affective disorder syndrome (SADS), in which symptoms usually start to appear in November and disappear in the late winter, after which many patients remain asymptomatic until the following fall (1, 2). Smaller seasonal variations in mood and behavior are also sometimes noted among patients with Depression, *per se*, but less so among normal control subjects (3, 4).

Multiple sclerosis (MS), an inflammatory neuroimmune disease which typically first affects women in their 20s, also often displays seasonality in the incidence of flare-ups: symptoms are more likely to occur in spring/summer than in fall/winter (5–7), and are more frequent in northern than in southern climates. The disease causes microglial activation, perhaps mediated by myelin-reactive T cells and specific IgG antibodies, which ultimately affects oligodendrocytes and their myelin product. The loss of myelin can lead to chronic neurodegeneration. The basic etiology of MS remains unknown; the disease has a genetic component (8) and has not been shown to involve viruses.

It should be noted that MS has recently become partly treatable with new drugs which, besides diminishing flare-ups, may modify its natural history: a large, recent (2017) 60-year population-based Norwegian study found that whereas median life expectancy of MS patients was significantly shortened (by about 7 years) among patients in the study’s initial time blocks, an effect on longevity was no longer observed in the last time block examined (1997–2012) (9), perhaps because partially effective treatments had started to become available.

## Seasonality of MS Flare-Ups

Several investigators have suggested that the propensity of MS attacks to occur most frequently in spring/summer is a consequence of concurrent reductions in plasma levels of the hormone melatonin (7, 10, 11) at that time of the year. These could be caused by the greater photic suppression of its synthesis (12) that occurs when daylight hours have been extended. A circadian rhythm in plasma melatonin among normal human subjects was identified by us in 1975 (13), with much higher levels occurring at nighttime than during the day. These circadian changes could underlie a circannual rhythm in peak or average plasma melatonin levels, or in the number of consecutive hours out of each 24-h period that the plasma levels are higher than their daily mean. Thus, peak daily melatonin levels, sampled in salivary samples obtained from Arctic urban residents, were highest in January and lowest in June when hours of ambient light are very great (14).

Disturbances in circadian melatonin rhythms commonly observed among shift workers (15) are also reportedly associated with an increased risk for MS (16): patients who did shift work for 3 years or longer prior to attaining age 20 significantly increased their vulnerability to MS, with odds ratios of 1.6; 95% CI, 1.2–2.1 in an incidence study (of new cases) and 1.3; 95% CI, 1.0–1.6 in a prevalence study (of total cases at steady state).

## Melatonin, Immune Mechanisms, and the Pathogenesis of MS

The activation of the pathologic processes underlying MS flare-ups reflects the balance between the actions of effector and regulatory T cells in lymphatic organs, brain, and other tissues (7). Melatonin blocks the differentiation and decreases the number of pathogenic Th17 T cells and slows the production of IL-17, a pathogenic cytokine. It also boosts the production of protective Tr1 cells and the cytokine IL-10 in non-pathogenic T cells.

Among animals with experimental autoimmune encephalitis (EAE), a widely used mouse model of MS, treatment with melatonin (5 mg/kg intraperitoneally, daily) was found by Farez et al. (7) to decrease the clinical symptoms of EAE and the number of T cells known to be associated with EAE (e.g., Th17 cells); it also slowed the formation of pathologic cytokines associated with the cells. Melatonin also boosted the formation of the protective Tr1 regulatory cells, and it or the melatonin analog agomelatine increased the expression by human CD4 T cells of the anti-EAE cytokine IL-10. To affirm the relevance of melatonin’s effects on T cells and their cytokines to human MS the investigators assessed, in serums from 26 MS patients, the correlations *in vivo* between melatonin levels and the expression of IL-17 and IL-10. As anticipated, higher serum melatonin levels were found to be negatively correlated with IL-17, and positively correlated with those of IL-10 (7).

Interestingly, melatonin’s suppressive effects on the pathogenic Th17 cells were found to be mediated by one of the two MTNR1A receptors (17, 18) that also mediates melatonin’s effects on CNS neurons (11). Moreover, in a clinical study, plasma melatonin levels were found to be negatively correlated with MS activity in a pool of 139 patients with relapsing-remitting MS who were followed over a fall and winter (7). In a smaller study (19), 8AM serum melatonin levels were significantly lower in MS patients than in control subjects.

It should be noted that no compelling evidence yet exists as to whether administering melatonin can modify the frequency or clinical manifestations of MS episodes; too few data are available to enable firm conclusions. As described above, a high daily intraperitoneal dose of melatonin (5 mg/kg) did ameliorate clinical symptoms in mice with EAE (7).

## Conclusion

Multiple Sclerosis exhibits several peculiarities among neurologic diseases relating to its temporal and gender relationships, any of which could provide useful hints about its pathogenesis or treatment. Its initial appearance tends to occur during the third decade of life; young women are more vulnerable to such attacks than young men; and attacks tend to be concentrated in the spring and summer seasons, and to regions closer to the North and South poles than to the equator. Since average blood melatonin levels tend to be lower at times and places where daylight is abundant for many hours each day, it is possible that these characteristics reflect a diminished ability of plasma melatonin to protect against the pathologic processes that promote MS (7). *In vitro* and animal studies provide evidence, discussed above, that melatonin can suppress the formation of T cells and cytokines that are pathogenic in MS and can stimulate formation of protective T cells and cytokines.

Perhaps it is time for systematic studies to be initiated to examine possibly useful effects of melatonin, or melatonin analogs, in preventing or treating MS flare-ups. If positive, the data would open a new chapter in melatonin physiology, in which the hormone would probably be administered not in the very small doses given as a “replacement therapy” to older people, whose calcified pineal glands secrete too little of the hormone to initiate and sustain nocturnal sleep (20), but in larger doses perhaps required to enable it to act as a drug (21).

## Author Contributions

RW, the sole author, conceptualized and wrote this manuscript.

## Conflict of Interest Statement

The author declares that the research was conducted in the absence of any commercial or financial relationships that could be construed as a potential conflict of interest.
